# Significant thrombus in saccular aneurysm in a patient with polycythemia: a case report

**DOI:** 10.1186/s13019-024-02645-7

**Published:** 2024-03-15

**Authors:** Atsuyuki Mitsuishi, Hiromasa Nakamura, Yujiro Miura, Tsuyoshi Miyata, Kazumasa Orihashi, Keisuke Yoshida, Kouhei Araki

**Affiliations:** 1 Department of Cardiovascular Surgery, Kochi Medical School Hospital, 185-1, Kohasu, Nankoku-shi, 783-8505, Okohcho, Kochi Prefecture Japan; 2 Liaison Healthcare Engineering Section Medicine, Kochi Medical School Hospital, 185-1, Kohasu, Nankoku-shi, 783-8505, Okohcho, Kochi Prefecture Japan; 3 Department of Anesthesiology and intensive Care Medicine, Kochi Medical School Hospital, 185-1, Kohasu, Nankoku-shi, 783-8505 Okohcho, Kochi Prefecture Japan; 4 Department of Surgery, Kochi Medical School Hospital, 185-1, Kohasu, Nankoku-shi, 783-8505, Okohcho, Kochi Prefecture Japan; 5 Department of Cardiovascular Surgery School of Medicine, Kochi University, 185-1, Kohasu, Nankoku-shi, 783- 8505, Okohmachi, Kochi Prefecture Japan

**Keywords:** Saccular aneurysm, Polycythemia, Mural thrombus, Peak wall stress, Endothelial cell, Rupture point, Vasa Vasorum, Case report

## Abstract

**Background:**

Morphologically, the risk of aortic aneurysm rupture is mainly evaluated based on its type (e.g., fusiform or saccular) and diameter. Based on the finite element analysis, peak wall stress has been identified as a more sensitive and specific predictor of rupture in recent years. Moreover, in finite analysis, the neck of aneurysm is the highest peak wall stress and is associated with the rupture point.

**Case presentation:**

A saccular aortic aneurysm (84 mm) was incidentally detected during preoperative examination for chronic empyema in a 74-year-old male patient with a history of polycythemia. Aortic arch graft replacement using an open stent was performed.

**Conclusions:**

Morphologically, this case was associated with a very high risk of rupture; nevertheless, it did not rupture. In this case, a mural thrombus (likely formed due to polycythemia) covered the neck of aneurysm that is experiencing the highest peak wall stress and is associated with the rupture point. The mural thrombus decreased peak wall stress and could reduce the risk of rupture even for huge saccular aneurysms. Furthermore, the mural thrombus was fully occupied in aneurysms, such as during coil embolization. Thus, polycythemia could decrease the risk of rupture of huge saccular aneurysms.

## Background

Morphologically, the risk of rupture is mainly evaluated based on the type (e.g., fusiform or saccular) and diameter. Reportedly, the wall stress based on finite element more accurately reflects the risk of rupture [1, 2]. Additionally, the neck of a saccular aneurysm is the point of highest peak wall stress and is associated with the rupture point [3, 4]. Moreover, the disappearance of endothelial cells is associated with a high risk of rupture [5]. Coil embolization is an intervention aiming to treat aneurysms not only by preventing blood flow from the aneurysm but also by preserving the intima. In this case, a mural thrombus (likely formed due to polycythemia) covered the aneurysm neck.

## Case presentation

The patient was a 74-year-old male who had suffered a cerebral infarction being diagnosed with secondary polythemia (JAK2V617F; Negative), who was scheduled for a surgery for maxillary cyst at the oral surgery department.

**Fig. 1:**
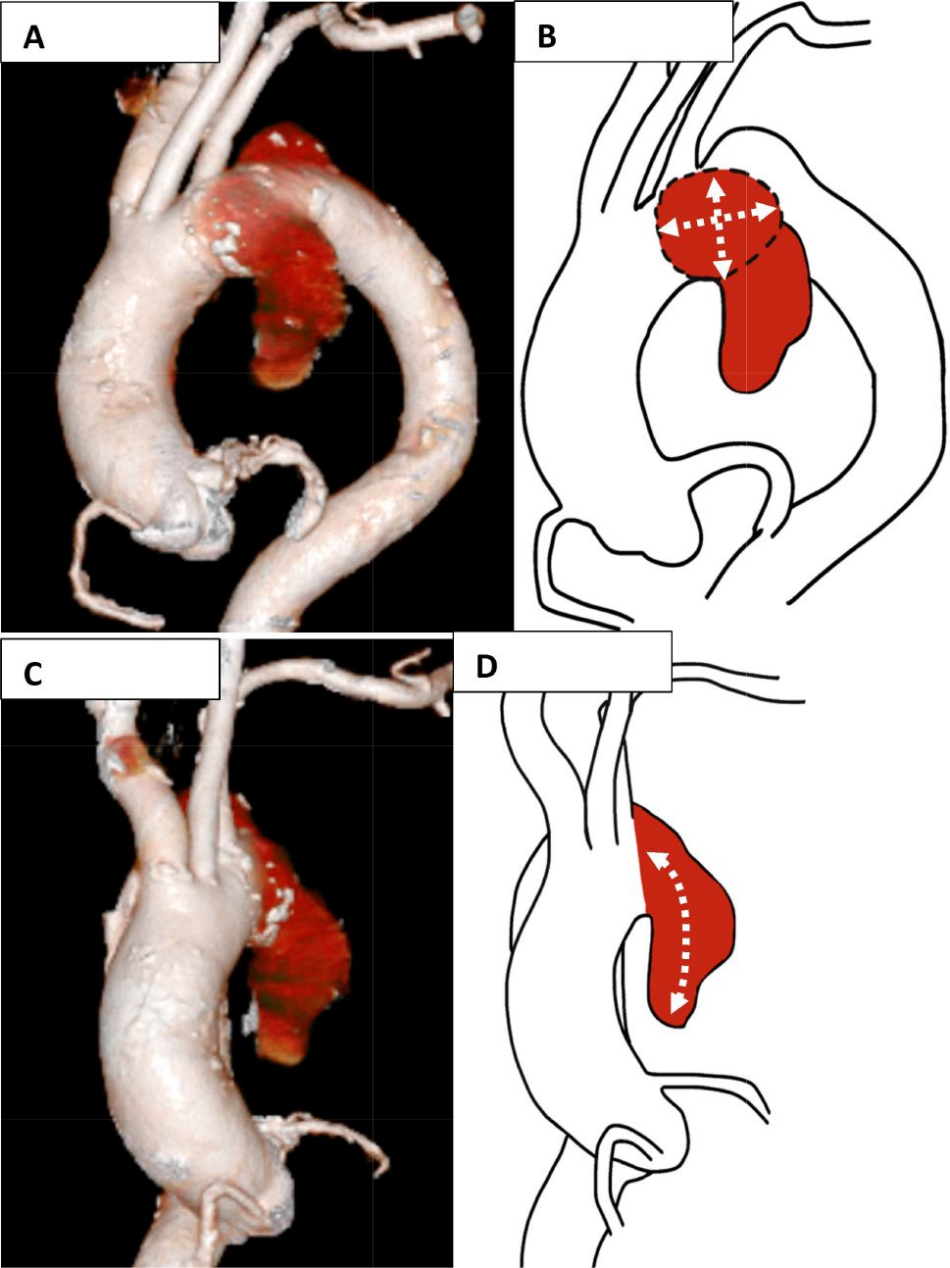
Preoperative 3D computed tomography. **A**, **B**: Front view. Saccular aneurysmal (Red color) width was 42 × 55 mm (White dashed arrowed). **C**, **D**: Left side view. Saccular aneurysmal neck was 84 mm (White dashed arrowed)

Preoperative examination revealed obstructive ventilation disorder; thus, the patient was referred to the respiratory department. His medication regimen at the time consisted solely of 100 mg of aspirin once a day. As part of the workup, a chest computed tomography (CT) angiography revealed a thoracic aortic aneurysm (84 mm). A saccular aneurysm protruded ventrally and caudally from the left subclavian artery to distal arch. The distance between the left carotid artery and proximal portion of the aneurysm was 11 mm. The depth of sac and width of neck were 42 × 55 and 84 mm, respectively (Fig. 1). Accordingly, the patient was referred to the cardiovascular department. His blood test showed hemoglobin 19.1 g/dL and hematocrit 54.9%; however, serum erythropoietin level was not measured (usually below the normal reference range in patients with polythemia vira) [6].

He was afebrile, with white blood cell count and C-reactive protein levels within the normal range, and negative blood culture results.

**Fig. 2:**
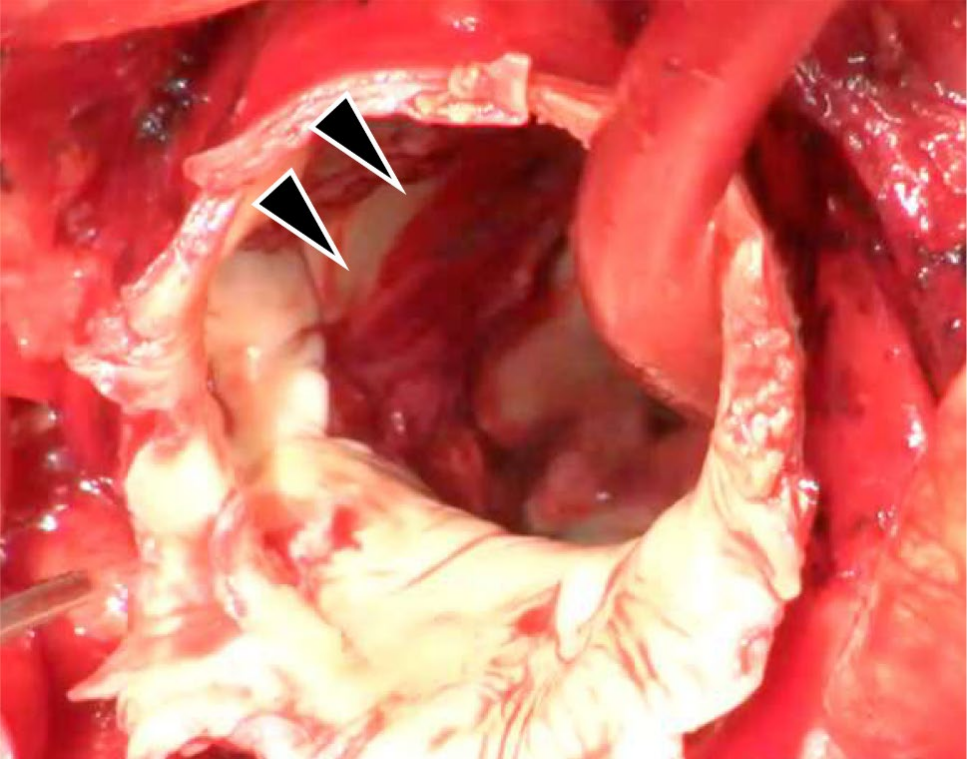
Intraoperative image. Mural thrombus surrounding the neck of the cystic aneurysm (Black arrows)

**Fig. 3:**
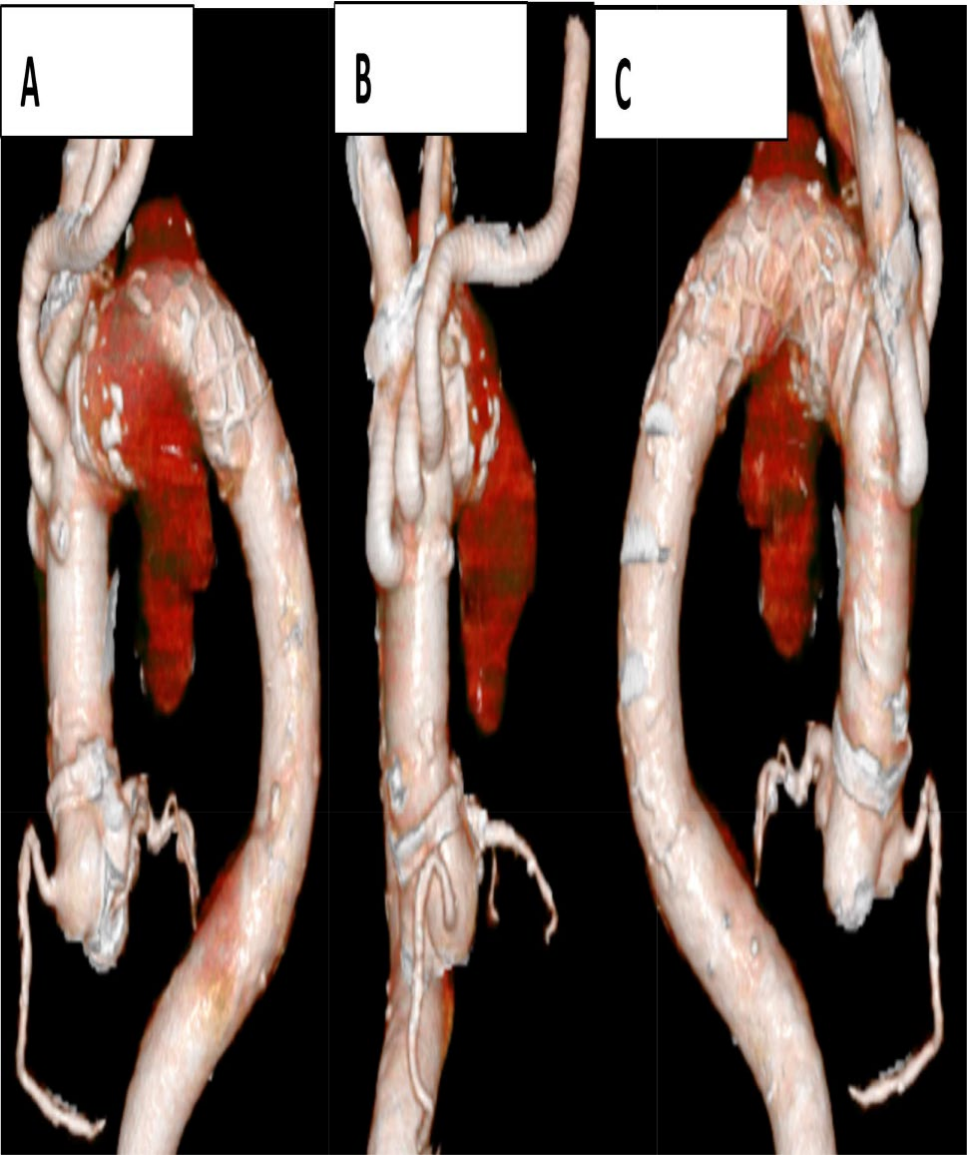
Postoperative 3D computed tomography

There was an aneurysm, which was 3 cm distal to the left subclavian artery, and to avoid distal anastomosis, we decided to perform an elective tortal arch replacement with a 29 mm ×90 mm frozen elephant trunk (J Graft FROZENIX; Japan Lifeline, Tokyo, Japan) surgery on the 26 mm aorta in Zone 4. Elective surgery was planned. Cardiopulmonary bypass (CPB) was established through median sternotomy with cannulations on the ascending aorta, and the superior and inferior vena cavae. The prosthetic graft was anastomosed to the left axillary artery. Bicaval drainage was established, and warm blood retrograde cardioplegia was administered every 20 min. We discontinued CPB and initiated selective cerebral perfusion from the left axillary artery at minimum laryngeal and bladder temperatures of 16.7 and 21.2 °C, respectively. Subsequently, we opened the aneurysm and placed cannulas (SP-GRIPFLOW™; Fuji Systems, Tokyo, Japan) in three carotid branches. Viewed from inside the aorta, the lumen of the saccular aneurysm was completely covered with a mural thrombus (Fig. 2). A frozen elephant trunk (J Graft FROZENIX; Japan Lifeline, Tokyo, Japan) was deployed under the guidance of a transesophageal echocardiogram. Following the completion of distal anastomosis with a 26-mm four-branch graft (Triplex; Terumo, Tokyo, Japan), anterograde perfusion of the lower body and rewarming were initiated through a graft branch. The duration of circulation arrest time to lower body perfusion was 55 min. The aneurysm contained a mural thrombus (Fig. 2). We transected the arch at zone 2 and reconstructed the left subclavian artery, left common carotid artery, and brachiocephalic artery. This was followed by a proximal anastomosis (Fig. 3). The total CPB and cardiac ischemic times were 240 and 128 min, respectively. On postoperative day 10, the patient was discharged without any neurological deficits or infection. After thoracic aortic aneurysm surgery, we aim to follow up with CT scans 1 week and 6 months after surgery. The next CT will be in 6 months.

## Discussion and conclusions

The prevalence of thoracic aorta aneurysm is ≤ 6–10 cases per 100,000 individuals [7]. Shang et al. [8] reported that saccular aortic aneurysms account for 1.5% of all aortic aneurysms. The causes of saccular aneurysm are trauma, infection, autoimmune disorders (e.g., Behcet’s disease or other collagen vascular diseases), or previous cardiovascular surgery [9].

However, the mechanism underlying the occurrence of saccular aneurysms has not been elucidated thus far.

Reportedly, the geometry of aortic arch is characterized by curvature and presence of major branches on the outer curvature. These characteristics play important roles in the development and natural course of saccular aortic aneurysms in this curved region [10]. In fact, cerebral arteries often branch at acute angles, and 90% of cerebral aneurysms are saccular aneurysms [11].

The most common site of saccular aneurysm occurrence is at the bifurcation of the anterior commissural artery. In this bifurcation, the antegrade blood pressure impinges on one side and, depending on the bifurcation angle, the wall stress is stronger. These characteristics increase the incidence of saccular aneurysms [12].

Morphologically, the risk of rupture is mainly evaluated based on diameter of the aneurysm. The relationship between aneurysm diameter and rupture is supported by Laplace’s law (Pierre-Simon Laplace, 1806). According to Laplace’s law, which states that the product of radius of curvature and pressure is equal to wall tension, for a given blood pressure, the larger aneurysm diameters are linked to greater wall stress.

However, in recent years, computational biomechanical modeling has been used to evaluate aortic wall stress (high aortic wall stress is associated with high risk of rupture) [1, 2] and wall shear stress (low wall shear stress is associated with high risk of rupture) [13] and possibly predict the behavior of aortic aneurysms for improvement of risk stratification [14, 15]. Based on the finite element analysis, a sac depth/neck width > 0.8 [13] and aspect ratio (vertical diameter/horizontal diameter) < 1.0 indicate a high risk of rupture [16]. However, although the aneurysm in the present case had a large diameter and was very prominent meeting conditions that a sac depth/neck width > 0.8 and aspect ratio < 1.0 in the neck, it did not rupture. The existence of such cases suggests the involvement of factors other than the diameter and type in aneurysm rupture.

Interestingly, in finite element analysis, the neck of the aneurysm shows the highest peak wall stress [1] and is associated with the rupture point [3, 4].

Surprisingly, in this case, the neck of the aneurysm was completely covered with mural thrombus, and the rupture point was located beneath that thrombus. Consequently, the neck of the aneurysm, which has the highest peak wall stress and has most likelihood of rupture, was covered by the mural thrombus. For mural thrombus, adhesion to the platelet wall is important, and the presence of red blood cells has a major influence on this, and it is known that the maximum effect is actually achieved at around 50% hematocrit [17, 18].

Under high hematocrit conditions (> 45% hematocrit), common in patients with polythemia, platelets are pushed centrifugally and are more likely to adhere to the vessel wall collagen and Von Willebrand factor, thus initiating thrombus formation [19]. High shear conditions resulting from vascular damage and elevated blood cell counts directly enhance the adhesion and activation of platelets [19]. In fact, there has been report mural thrombus in a patient with polycythemia [20]. In this case, the formation of a mural thrombus in the saccular aneurysm was potentially attributed to high hematocrit due to polycythemia and vascular damage in saccular aneurysm.

Furthermore, in this case, the mural thrombus may have formed a thrombus within the aneurysm while maintaining intima similar to the coil embolization of aneurysm [21, 22].

This may also prevent rupture because the walls of ruptured saccular aneurysms are characterized by loss of endothelial cells [5]. The combination of endothelial cell loss and proteolytic injury by wall stress predisposes the saccular aneurysm wall to rupture. Hence, the loss of endothelial cells is a key event that leads to the degeneration and eventual rupture of the saccular aneurysm wall.

In addition, it has been reported that the disappearance of endothelial cells introduced by blood flow and conversion into a pseudoaneurysm are associated with a high risk of rupture. Moreover, the occurrence of intima hyperplasia reduces the risk of aneurysm rupture [5].

On the contrary, the presence of a mural thrombus reduces blood flow from within the blood vessel to the wall, causing ischemia and weakening the blood vessel wall [5]. Indeed, very high endogenous peroxidase activity is observed in peroxidase-stained samples of saccular aneurysm walls with a luminal thrombus [23]. Furthermore, the presence of adherent inflammatory mononuclear cells on the endothelium indicates dysfunction of the endothelium in this area. Dysfunction may be associated with impaired rheology in this vasa vasorum vessel. Violation of the blood circulation to the aortic wall causes media necrosis and contributes to the development of aortic aneurysm dissection [24].

However, in this case, mural thrombus was promoted by polycythemia, and location in the thoracic region, with abundant vasa vasorum [25], might have weakened the effects of ischemia.

In addition, it has been reported that patients with a mural thrombus have a lower peak wall stress and a lower risk of rupture than those without a wall thrombus [2].

In this case, a mural thrombus (likely formed due to polycythemia) led to vascular necrosis and formation of a huge aneurysm; however, the mural thrombus covered the neck of aneurysm as a rupture point, effect of preserving intima like coil embolization and decrease in peak wall stress may be possible to reduce the rupture risk even for huge saccular aneurysms. Moreover, the aneurysm was formed in the thoracic aorta, where the vasorum is abandoned leading to reduction of the ischemic effect.

It would be desirable to evaluate not only the aneurysm diameter and type, but also peak wall stress, presence of an intramural thrombus covering the neck of the aneurysm, tissue changes (i.e., presence or absence of endothelial cells), and development of the vasa vasorum. This approach would allow physicians to make judgments based on more multifaceted findings and, consequently, perform a more accurate assessment of the risk of rupture.

There are some limitations in this study. Firstly, the actual peak wall stress around the aneurysm in this case is unknown. Secondly, due to deployment of a frozen elephant trunk in Zone 2, the stent was placed over the saccular aneurysm and aneurysm wall could not be excised as an intraoperative specimen. Thus, whether endothelial cells remained in the aneurysm remains unknown. Thirdly, it is difficult to evaluate endothelial cells and vasa vasorum preoperatively. In the future, it would be beneficial if we could use modalities such as CT or magnetic resonance imaging for evaluation. Fourth, the fact the huge saccular aneurysm did not rupture does not ensure that it may not rupture in due time. Finally, this is a case report; thus, verification of the findings in a study involving a larger population is required in the future.

## Data Availability

All data generated or analyzed during this study are included in this article.

## Abbreviations

CPB: cardiopulmonary bypass
CT: computed tomography
